# Purification and Molecular Characterization of Fucoidan Isolated from *Ascophyllum nodosum* Brown Seaweed Grown in Ireland

**DOI:** 10.3390/md21050315

**Published:** 2023-05-21

**Authors:** Gaurav Rajauria, Rajeev Ravindran, Marco Garcia-Vaquero, Dilip K. Rai, Torres Sweeney, John O’Doherty

**Affiliations:** 1 School of Microbiology, School of Food and Nutritional Sciences, SUSFERM Fermentation Science and Bioprocess Engineering Centre, University College Cork, T12 K8AF Cork, Ireland; grajauria@ucc.ie; 2 Circular Bioeconomy Research Group, Shannon Applied Biotechnology Centre, Munster Technology, V92 CX88 Tralee, Ireland; rajeev.ravindran@mtu.ie; 3 School of Agriculture and Food Science, University College Dublin, Belfield, D04 V1W8 Dublin, Ireland; marco.garciavaquero@ucd.ie; 4 Department of Food Biosciences, Teagasc Food Research Centre, Ashtown, D15 KN3K Dublin, Ireland; dilip.rai@teagasc.ie; 5 School of Veterinary Medicine, Veterinary Science Centre, University College Dublin, Belfield, D04 V1W8 Dublin, Ireland; torres.sweeney@ucd.ie

**Keywords:** fucoidan, macroalgae, antioxidant activity, Fourier transform infrared (FTIR), quadruple time of flight mass spectrometry (Q-ToF-MS), molecular weight cut-off filter (MWCO), solid-phase extraction (SPE)

## Abstract

The present study investigates the molecular characteristics of fucoidan obtained from the brown Irish seaweed *Ascophyllum nodosum*, employing hydrothermal-assisted extraction (HAE) followed by a three-step purification protocol. The dried seaweed biomass contained 100.9 mg/g of fucoidan, whereas optimised HAE conditions (solvent, 0.1N HCl; time, 62 min; temperature, 120 °C; and solid to liquid ratio, 1:30 (*w*/*v*)) yielded 417.6 mg/g of fucoidan in the crude extract. A three-step purification of the crude extract, involving solvents (ethanol, water, and calcium chloride), molecular weight cut-off filter (MWCO; 10 kDa), and solid-phase extraction (SPE), resulted in 517.1 mg/g, 562.3 mg/g, and 633.2 mg/g of fucoidan (*p* < 0.05), respectively. In vitro antioxidant activity, as determined by 1,1-diphenyl-2-picryl-hydrazyl radical scavenging and ferric reducing antioxidant power assays, revealed that the crude extract exhibited the highest antioxidant activity compared to the purified fractions, commercial fucoidan, and ascorbic acid standard (*p* < 0.05). The molecular attributes of biologically active fucoidan-rich MWCO fraction was characterised by quadruple time of flight mass spectrometry and Fourier-transform infrared (FTIR) spectroscopy. The electrospray ionisation mass spectra of purified fucoidan revealed quadruply ([M+4H]^4+^) and triply ([M+3H]^3+^) charged fucoidan moieties at *m/z* 1376 and *m/z* 1824, respectively, and confirmed the molecular mass 5444 Da (~5.4 kDa) from multiply charged species. The FTIR analysis of both purified fucoidan and commercial fucoidan standard exhibited O-H, C-H, and S=O stretching which are represented by bands at 3400 cm^−1^, 2920 cm^−1^, and 1220–1230 cm^−1^, respectively. In conclusion, the fucoidan recovered from HAE followed by a three-step purification process was highly purified; however, purification reduced the antioxidant activity compared to the crude extract.

## 1. Introduction

Seaweed or macroalgae are a remarkable group of organisms capable of faster production/farming cycles and higher resource efficiency when compared to other terrestrial plants [1]. Moreover, seaweed offers great biodiversity, with over 2000 brown macroalgal species described to date [2]. These marine plants have a greater capacity to adapt and survive changes in the marine environment by the production of chemical compounds, different in composition to those of terrestrial plants, that can be potentially exploited as new pharmaceuticals, cosmeceuticals, and functional foods as they display a wide range of biological activities [3].

Polysaccharides are the most widely produced compounds by brown seaweed, reaching levels that can vary between 4 and 76% [3]. Thus, brown seaweeds, such as *Ascophyllum nodosum*, are currently exploited as a source of hydrocolloids, mainly fucoidan, laminarin, and alginates, that have been widely used by the food industry as thickening, gelling, or film-forming agents due to their physicochemical properties [4,5]. Recently, fucoidan has been also explored for other purposes due to the wide range of biological activities of these molecules, such as antioxidant, antitumour, antimicrobial, and immunostimulatory activities, amongst others, when assayed in vitro and/or in vivo [6,7,8].

Chemically fucoidan or fucose-containing sulphated polysaccharides are a group of compounds of varied molecular sizes ranging from 10–10,000 kDa [8]. These polysaccharides are generally described as having a backbone of (1 → 3)-α-l-fucopyranosyl or alternating (1 → 3) (1 → 4)-α-l-fucopyranosyl residues and variable levels of branching (1 → 6)-β-d-galacto- and/or (1 → 2)-β-d-mannopyranosyl units with fucose or fuco-oligosaccharide branching, and/or glucuronic acid, xylose, or glucose substitutions [9,10]. Moreover, the l-fucopyranose residues may have sulphate substitutions on C-2, C-4, and C-3 positions [9,10]. These wide variations in the chemical structure of fucoidan also have an influence on the biological activities of the compounds and thus, their prospective industrial applications. Understanding the chemical structure of fucoidan requires a multi-actor approach due to the wide variety of factors influencing both the abundance and chemical structure of these molecules. The abundance and chemical composition of polysaccharides and other bioactive compounds produced by seaweed can be affected by intrinsic factors affecting the biomass, including the macroalgal species, reproductive stage of the biomass, and/or parts of the seaweed sampled, but also season and place of collection that influence the abiotic conditions in which the biomass grows [11,12]. Moreover, the commercial exploitation of macroalgal polysaccharides requires the extraction of these compounds from the biomass, by using conventional and/or innovative techniques at variable extraction conditions (i.e., solvent, time, temperature, and pressure) aiming to extract high yields of fucoidan [13,14]. These extraction processes, independent of the extraction technology used, have a significant influence on the yields of compounds, their chemical structure, and their biological properties. There are several reports on the use and optimization of extraction technologies aiming to achieve high yields of fucoidan from brown seaweed [15] and more recently, several studies have switched this approach to focus on a combined optimization of yields and biological properties in vitro, to ensure that these characteristics are preserved during the processing of the biomass [14,15]. Further processing of the fucoidans, including steps of filtration and one or several chromatographic separation techniques for the purification of these compounds may also influence the chemical structure of fucoidan and the biological properties of the purified molecules [16,17]. Thus, there is a need to develop procedures involving sequential and systematic studies approaching this issue, including analysis of factors influencing the variability of the composition of brown seaweed, the influence of the extraction processes on composition and biological properties of the macroalgal compounds in vitro and in vivo, and final elucidation of the chemical structure of the active fucoidan responsible for these effects. The development of these studies will be key to gathering essential knowledge on this molecule to allow the development of good manufacturing practices that will help in the official process of approval of fucoidan and their derived fractions for multiple industrial applications, including its use in pharmaceutical and nutraceutical sectors [6].

Prior to this study, the seasonal variation and influence of climatological parameters on the abundance of fucoidan and other bioactive compounds produced by the Irish brown seaweed *A. nodosum* was analysed [11]. A new hydrothermal-assisted extraction process was developed and optimised for the extraction of high yields of fucoidan while monitoring the antioxidant activities in vitro of the compounds achieved from *A. nodosum* [18] and further evaluation of the antimicrobial and probiotic potential of these fucoidan extracted from *A. nodosum* using the newly developed methods were analysed both in vitro and in vivo [19,20]. The current study aims to develop an efficient purification procedure involving molecular weight cut-off and solid phase extraction for fucoidan extracted from *A. nodosum*, monitoring the purification process by measuring the yields of fucoidan and their antioxidant activities in vitro, as well as the elucidation of the chemical structure of fucoidan by electrospray ionization mass spectrometry (ESI-MS) and Fourier-transform infrared (FTIR) spectroscopy.

## 2. Results and Discussion

### 2.1. Biochemical Composition Analysis

Biochemical composition analysis of dried whole *A. nodosum* seaweed biomass and the crude extract was conducted to determine the composition of the different components in the matrix. The biochemical composition of dried whole seaweed biomass reported earlier [11] contained fucoidan (100.9 mg/g seaweed), total glucan (27 mg/g seaweed), total soluble sugars (136.6 mg/g seaweed), crude protein (61.4 mg/g seaweed), ether extract (33.3 mg/g seaweed), total polyphenols (6.7 mg/g seaweed), and ash (233.1 mg/g seaweed) (Table 1). The availability of a high fucoidan content makes this seaweed species a potential source for fucoidan production, an observation that agrees with previous reports [21,22].

The biochemical composition of the fucoidan-rich crude extract produced by HAE is presented in Table 2. The composition analysis revealed that the crude extract was rich in fucoidan (417.6 mg/g dried extract) followed by laminarin (165.8 mg/g dried extract), mannitol (96.0 mg/g dried extract), and alginate (41.4 mg/g dried extract). The crude extract also contained crude protein (40.3 mg/g dried extract) and ash (6.7 mg/g dried extract). Previous studies reported that the maximum recovery of polysaccharides from seaweed can be achieved at higher temperatures [23,24,25,26]. It is also evident in this study as 54.4% of fucoidan in the total composition amount of the crude extract was recovered at 120 °C using HAE. Fucoidans are storage sulphated polysaccharides localized in the cell walls of brown seaweeds, the cell walls need to be hydrolyzed or broken down to release these biomolecules into the extraction media. Cell wall disruption can be achieved by various methods, such as enzymatic digestion, acid hydrolysis, and mechanical disruption using agitation or physical forces like ultrasound, microwaves, hydrothermal, or high pressure [24,27,28]. Several studies have been reported that employ various technologies for the extraction of fucoidan from seaweed. For instance, Okolie et al. [21] extracted fucoidan from *A. nodosum* using conventional chemical methods and advanced microwave-assisted, ultrasound-assisted, and enzyme-assisted extraction methods. The authors reported the highest extraction yield of fucoidan from conventional chemical (11.9%) and microwave-assisted (5.71%) extraction methods. In another study, fucoidan was extracted from *Sargassum mcclurei* brown seaweed by an ultrasound-assisted extraction method using ethanol and reported a yield of 7.1 mg/g of total polysaccharide content (representing fucoidan) in the extract [29].

### 2.2. Purification of Fucoidan

A multi-step (three-step) approach for the purification of fucoidan was undertaken. The first step involved the removal of proteins. Protein removal from seaweed matrices is conventionally achieved by the application of mild alkaline and acidic conditions while employing distilled water as the extraction solvent. A combination of low temperatures (in the range of 4 °C) and centrifugation measures are used for this purpose [30]. However, in this study, proteins from the crude extract were precipitated by 100% ethanol and removed by centrifugation. The precipitation step was carried out by incubating samples overnight at 4 °C to facilitate the maximum removal of protein from the extract. After that, the removal of any residual alginate was carried out by employing CaCl_2_ [23]. Alginate reacts with calcium for an insoluble precipitate which can be removed by centrifugation or filtration [31]. Ethanol can then be employed to wash the pellet obtained as this will lead to the solubilisation of calcium alginate and its subsequent removal. This technique eliminates the need for hazardous alkaline and acidic conditions which have been employed in certain studies [32]. Alginate analysis of the pellet obtained revealed 0.089 g of alginate per g of dry matter, which amounted to 90% removal.

The alginate-free extract was resuspended in 500 mL of 75% ethanol (*v/v*) and centrifuged at 8000 rpm for 10 min to remove any residual protein. The pellet was redissolved in 100 mL of ultrapure water and subjected to ultrafiltration employing centrifugal concentrators (MWCO; 10 kDa). The fucoidan-rich fraction was collected, freeze-dried, redissolved in 0.1 HCl, and passed through solid phase extraction cartridges. Fucoidan is a negatively charged molecule with hygroscopic properties; therefore, Strata^®^ SI-1 Silica, which has been tested for the separation of polar analytes, was employed for the separation of fucoidan.

### 2.3. Quantification of Fucoidan

The efficacy of the purification process was analysed by quantification of fractions obtained after solvent extraction, ultrafiltration, and solid phase extraction employing HPLC. Retention times for the three saccharides viz. fucoidan, laminarin, and mannitol were observed to be 16.684 min, 17.659 min, and 21.509 min, respectively. Attempts to quantify the polysaccharides in the crude extract did not result in satisfactory outcomes, possibly due to the interference of other components, such as alginate. The concentration of each polysaccharide in the different purified fractions is presented in Table 3. Interestingly, all three fractions contained high amounts of fucoidan, as compared to laminarin and mannitol. However, the fucoidan content in the extract increased progressively with each purification step. Solid-phase extraction resulted in the highest yield of fucoidan in the SPE fraction (633.23 ± 0.03 mg/g of dried extract). Additionally, laminarin (124.2 ± 0.02 mg/g of dried extract) and mannitol (76.45 ± 0.05 mg/g of dried extract) were also found in small amounts in the same fraction. Meanwhile, the solvent purified fraction contained the lowest amount of fucoidan (517.12 ± 0.02 mg/g of dried extract) along with laminarin (172.2 ± 0.01 mg/g of dried extract) and mannitol (112.5 ± 0.02 mg/g of dried extract).

### 2.4. Antioxidant Properties of Fucoidan-Rich Purified Fractions

The DPPH free radical scavenging activity assay is a very simple, rapid, and effective method to measure the antioxidant capacity of a molecule. The DPPH^•^ in methanol solution (a stable diamagnetic molecule) is reduced to DPPH-H, a non-radical form in the presence of an antioxidant that can donate hydrogen. The antioxidant capacity of the substance would be measured as its ability to scavenge DPPH^•^ free radical. All the fucoidan fractions were subjected to DPPH radical scavenging activity. The DPPH scavenging activity of the crude extract, the fucoidan fractions obtained after each purification step, and commercial fucoidan and ascorbic acid are presented in Table 4. All the fractions exhibited antioxidant activities in various capacities. The crude extract (43.0%; IC_50_ = 1.16 mg/mL) showed the highest antioxidant capacity (*p* < 0.05) compared to the purified fucoidan fractions (22.6–37.8%), commercial fucoidan (21.7%; IC_50_ = 2.30 mg/mL), and ascorbic acid standard (35.2%; IC_50_ = 1.42 mg/mL). These values were comparable with literature available elsewhere. For example, Chen, et al. [33] tested the DPPH scavenging activity of four fractions of fucoidan obtained from *A. nodosum* and reported values in the range of 45.9%, 30.4%, 32.5%, and 98.2%, respectively. Athukorala et al. [34] reported that the scavenging activity of methanolic extract of red algae *Grateloupia filicina* (65%) was over two-fold that of butylated hydroxy toluene (BHT) and a-tocopherol (22% and 32%). Similarly, Lekameera et al. [35] reported that the DPPH scavenging activity of the methanolic extract of brown algae *Colpomenia sinuosa* was higher (88.57%) than that of commercial antioxidants butylated hydroxyl anisole (87.38%) and BHT (56.05%) at 2 mg/mL concentration. In the current study, composition analysis of the crude extract revealed that it was rich in polyphenols in addition to fucoidan and laminarin. The polyphenolic content of seaweed significantly contributes to its antioxidant properties. This may have resulted in high DPPH radical scavenging activity of the crude extract. As the purification of the crude extract progressed, the antioxidant activity of the purified fractions reduced. Among the purified fractions, the solvent purified fraction exhibited the highest (*p* < 0.05) DPPH radical scavenging capacity (37.8%; IC_50_ = 1.32 mg/mL) followed by the MWCO fraction (23.1%; IC_50_ = 2.16 mg/mL) and the SPE fraction (22.6%; IC_50_ = 2.21 mg/mL) (Table 4).

In an acidic pH of 3.6 the solubility of iron increases resulting in reduced ionisation potential. This drives hydrogen ion transfer and subsequent redox reaction dominant mechanism. During the FRAP assay, the ability of a molecule to transfer an electron to Fe^3+^ ions to reduce it to Fe^2+^ ions is measured. For the reasons mentioned above, this assay is conducted at an acidic pH. The reduction of Fe^3+^ to Fe^2+^ gives rise to a change in colour which can be measured spectrophotometrically at 593 nm. The FRAP assay is a preferred method for the measurement of the antioxidant capacity of a particular compound due to the fast nature of the reaction [36]. The FRAP assay was conducted on the crude extract, commercial fucoidan, and purified fucoidan fractions to study the effect of the purification strategy. The antioxidant capacity of crude extract and purified fucoidan fractions to reduce the TPTZ–Fe (III) complex to the TPTZ–Fe (II) complex is presented in Table 4. Observations comparable to the DPPH assay were detected for the crude extract and purified fractions; a steady decline in the FRAP value was registered with the progression of the purification strategy. The highest FRAP activity was registered by the crude extract (38.63 ± 0.2 mg TE/g) which can again be attributed to the presence of high concentrations of polyphenols in the same extract. This was followed by the solvent purified fraction (30.8 ± 0.4 mg TE/g), the MWCO fraction (14.8 ± 0.5 mg TE/g), and the SPE fraction (14.5 ± 0.3 mg TE/g). A sharp decline in the FRAP value was observed between the solvent fraction and the succeeding MWCO and SPE fractions. However, no significant difference was observed in the FRAP values of the MWCO fraction and the SPE fraction. Additionally, the FRAP value of the crude extract and purified fractions were higher than the commercial fucoidan standard employed (13.9 ± 0.7 mg TE/g). Several studies have been conducted on the FRAP capacities of *A. nodosum* extracts. For example, Agregán, et al. [37] reported FRAP values for *A. nodosum* extracts to be in the range of 4.40–4.66 µmol TE/g DW. Variations in observations may be attributed to several factors ranging from extraction procedures to harvest conditions.

It is worth noting that while the highest fucoidan content (*p* < 0.05) was recorded in the SPE fraction (Table 3) but no significant difference in antioxidant activity of the SPE fraction and the MWCO fraction measured by DPPH scavenging capacity and FRAP assay was observed (Table 4). Thus, the MWCO fraction which contained higher fucoidan content and antioxidant capacity was selected for molecular characterisation.

### 2.5. Structural Characterisation of Purified Fucoidan by ESI-MS

**Figure 1:**
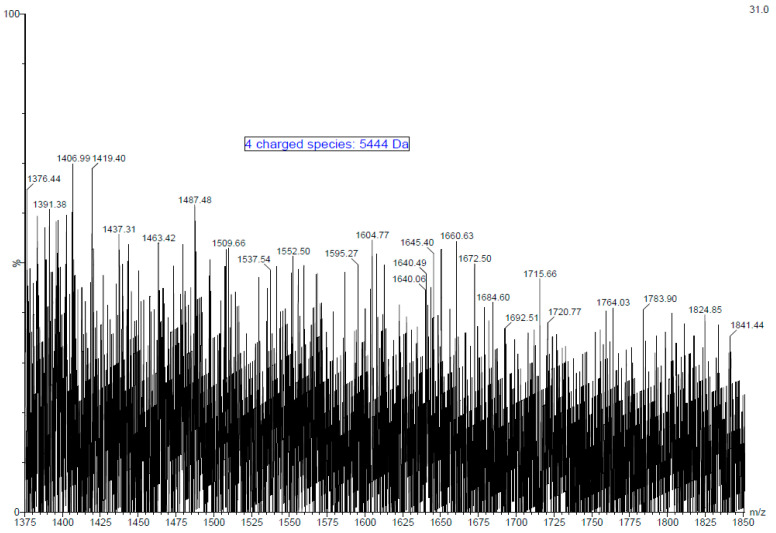
Electrospray ionisation mass spectra (ESI-MS) of purified fucoidan obtained from MWCO Fraction from *A. nodosum* seaweed.

Fucoidan is a large molecule with polydispersity and high molecular weight. The electrospray ionisation mass spectra (ESI-MS) of fucoidan obtained from the MWCO fraction of *A. nodosum* was measured and a typical mass spectrum of the fucoidan molecule is presented in Figure 1. Signals of high intensity were obtained as expected due to the presence of sulphate groups. Furthermore, numerous peaks distributed throughout the spectrum reveal the heterogeneous nature of the fucoidan molecule. Furthermore, the complexity of the spectrum is augmented by the ability of the poly-sulphated oligosaccharide to form multiply charged ions. The oligosaccharide size ranged from mono to tetrafucose with usually one sulphate group per fucosyl unit. Monosulphated fucose units can be identified by at *m/z* of 243 [M-H]^−^, whereas disulphated fucose is represented by *m/z* of 161 [M-2H]^2−^ by negative ion mode [38]. The presence of species such as trisulphated trifucose and tetrasulphated tetrafucose was not identified probably due to the bulky nature of the molecule and the presence of impurities. Quadruply ([M+4H]^4+^) and triply ([M+3H]^3+^) charged fucoidan moieties were identified by *m/z* peaks at 1376 and 1824, respectively. The molecular mass of the fucoidan molecule purified from *A. nodosum* was determined to be 5444 Da (~5.4 kDa) based on these multiply charged species. Most literature available on fucoidan deals with a low molecular weight form of the polysaccharide. For example, Li, et al. [39] reported the extraction of fucoidan from *A. nodosum* with a molecular weight of 3090 Da. Recently, Okolie et al. [21] extracted fucoidan from *A. nodosum* using a number of extraction methods and reported molecular weight values of 2.7 kDa–136.3 kDa. The difference in the molecular weight of fucoidan is mainly attributed to seaweed species, harvesting location, seasonal variation, climatic conditions, and extraction/purification method used [10,40,41]. Several studies that involve the characterisation of fucoidan by mass spectroscopy perform hydrolysis of the molecule. However, hydrolysis steps were overlooked, as the morale behind performing mass spectroscopy of the purified fucoidan was to identify its molecular weight and thus beyond the scope of this work. The molecular weight of seaweed purified polysaccharides plays a key role in biological activity, and this was the main focus in determining the same [42]. For example, in a recent study, Miyazaki, et al. [43] reported that high molecular weight fucoidan induces anti-tumour immune function in cells. In another significant study, researchers were able to successfully restore the natural killer cell cytotoxicity and granzyme B in addition to increasing the expression of interleukins and tumour necrosis factor in immunosuppressed mice with the aid of high molecular weight fucoidan derived from *Undaria pinnatifida* [44].

### 2.6. Characterisation of Purified Fucoidan Using FTIR Spectroscopy

**Figure 2:**
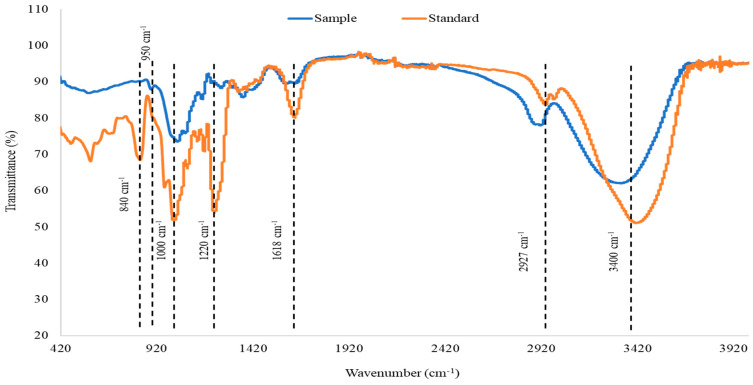
FTIR spectra of purified fucoidan obtained from MWCO Fraction from *A. nodosum* (purified test sample) in comparison with commercial fucoidan standard (from Sigma).

The purified MWCO Fraction was further analysed by employing FTIR spectroscopy. The FTIR spectra of the purified fucoidan and commercial fucoidan from *F. vesiculosus* are presented in Figure 2. Both sample and commercial standard exhibited O-H stretching and C-H stretching which are represented by bands at around 3400 cm^−1^ and 2920 cm^−1^, respectively. However, the C-H stretching was more prominent in the fucoidan obtained from *A. nodosum* compared to the standard. The peak at 1618 cm^−1^ signifies carbonyl groups that form uronic acid. The intensity of this peak was significantly higher in the standard as compared to the control. Uronic acid is one of the building blocks of fucoidan, and the levels of this component may vary based on the seaweed species [45]. Fucoidan contains ester sulphate which is detected in the IR spectra as a peak at 1220–1230 cm^−1^ which is attributed to S=O stretching. Fucoidan extracted from *A. nodosum* appeared to have a lesser amount of ester sulphate compared to the standard. Transmittance at 1000–1100 cm^−1^ is an indication of the presence of polysaccharides exhibited by C-O stretching. In fucoidan, this peak is contributed by the presence of glucuronic acid which is another component that forms the polysaccharide. A left shift in this peak indicates the presence of short chains that are highly polar. Such an observation may be made for fucoidan which was purified from *A. nodosum.* The presence of glucuronic acid may be the underlying reason for the high polarity. Peaks at 840 cm^−1^ and 850 cm^−1^ are indicative of sulphate groups in fucoidan. Commercial fucoidan standard exhibited a peak at 840 cm^−1^. However, this peak was absent in fucoidan purified from *A. nodosum.* Instead, a small peak was observed at 950–960 cm^−1^ which showed the presence of the asymmetrical stretching vibration of C–O–S bonds.

## 3. Materials and Methods

### 3.1. Chemicals and Reagents

Sulphuric acid, hydrochloric acid, ethanol, methanol, sodium tetraborate, carbazole, cysteine hydrochloride, ferric chloride, sodium phosphate dibasic, triton™ X-100, and potassium hydroxide were purchased from Sigma (Sigma-Aldrich, St. Louis, MO, USA). Acetic acid and sodium hydroxide were purchased from VWR (VWR International, Radnor, PA, USA). Standards such as Trolox (6-hydroxy-2,5,7,8-tetramethylchromane-2-carboxylic acid), DPPH (1,1-diphenyl-2-picryl-hydrazyl), TPTZ (2,4,6-tripyridyl-s-triazine), ascorbic acid, alginic acid, mannitol, and commercial fucoidan (from *Fucus vesiculosus*), and laminarin (from *Laminaria digitata*) standards were also purchased from Sigma. Enzyme-based kits for mannitol and glucan assay were purchased from Megazyme International Ltd., (Bray, Ireland). All reagents used were of analytical grade.

### 3.2. Seaweed Biomass and Chemical Composition Analysis

*A. nodosum* was harvested and supplied by Quality Sea Veg Ltd. (Burtonport, Co. Donegal, Ireland) in February 2016. The seaweed samples were cleaned, oven-dried (50 °C for 9 days), and then milled to a 1 mm particle size using a hammer mill (Christy and Norris, Chelmsford, UK). The milled samples were vacuum-packed and stored at −20 °C for further analyses. The seaweed samples were collected in bulk in the years 2016 and 2017. The selection of February 2016 samples for fucoidan extraction is based on the findings of our extensive seasonal variation study published earlier [11]. Chemical composition analysis including dry matter, ash, and ether extract was determined according to the AOAC methods [46,47,48]. The protein content was measured by the LECO TruSpec instrument (LECO Instruments UKLTD., Cheshire, UK) by determining the N content and multiplying it with the conversion factor of 4.17 reported for brown seaweed [49]. The total soluble sugars were estimated following the phenol-sulfuric acid assay (Brummer and Cui, 2005), whereas total glucans were measured by employing a K-YBGL kit (Megazyme, Bray, Ireland) following the manufacturer’s recommendations. Fucoidan was estimated by methodology devised by Usov, Smirnova, and Klochkova, [50]. Briefly, while keeping the mixture in ice, 1 mL of the sample was mixed with 4.5 mL of conc. H_2_SO_4_ (1:6, *v/v*). The mixture was then brought to room temperature (24 °C) following which it was incubated in an actively boiling water bath for 10 min. After cooling the tubes, 0.1 mL of 3% cysteine hydrochloride was added and the mixture was allowed to stand for 1 h until it turned yellow. Fucose was used as standard.

### 3.3. Hydrothermal-Assisted Extraction of Fucoidan

Hydrothermal-assisted extraction of fucoidan from *A. nodosum* was performed by applying optimised extraction conditions and the procedure reported in our earlier work [51]. Briefly, dried and milled seaweed was suspended in 0.1N HCl maintaining a solid-to-liquid ratio of 1:30 (*w/v*). The mixture was thoroughly agitated to ensure uniformity and then subjected to a thermal treatment at 120 °C for 62 min in an autoclave (Tomy SS-325; Tomy Seiko Co. Ltd., Tokyo, Japan). The extraction was performed in duplicate, and solids were separated from the liquids using the Whattman^®^ number 3 (Sigma-Aldrich, St. Louis, MO, USA). The crude liquid extract was used for a sequential purification process, and the purified fractions were then freeze-dried and used for antioxidant activity analysis. A portion of the crude liquid extract was freeze-dried and screened for biochemical composition analysis.

### 3.4. Biochemical Composition Analysis of Fucoidan-Rich Crude Extract

The fucoidan-rich crude extract was tested for biochemical compositions. Fucoidan, dry matter, ash, and protein content in the crude extract was estimated according to methods described in Section 2.2. Laminarin and mannitol were quantified by using K-YBGL and K-MANOL standard kits from Megazyme (Megazyme, Bray, Ireland), respectively. The alginate content in the crude extract was estimated according to the protocol reported by Truus, Vaher, and Taure [52]. Alginic acid was used as standard.

### 3.5. Purification of Fucoidan

**Figure 3:**
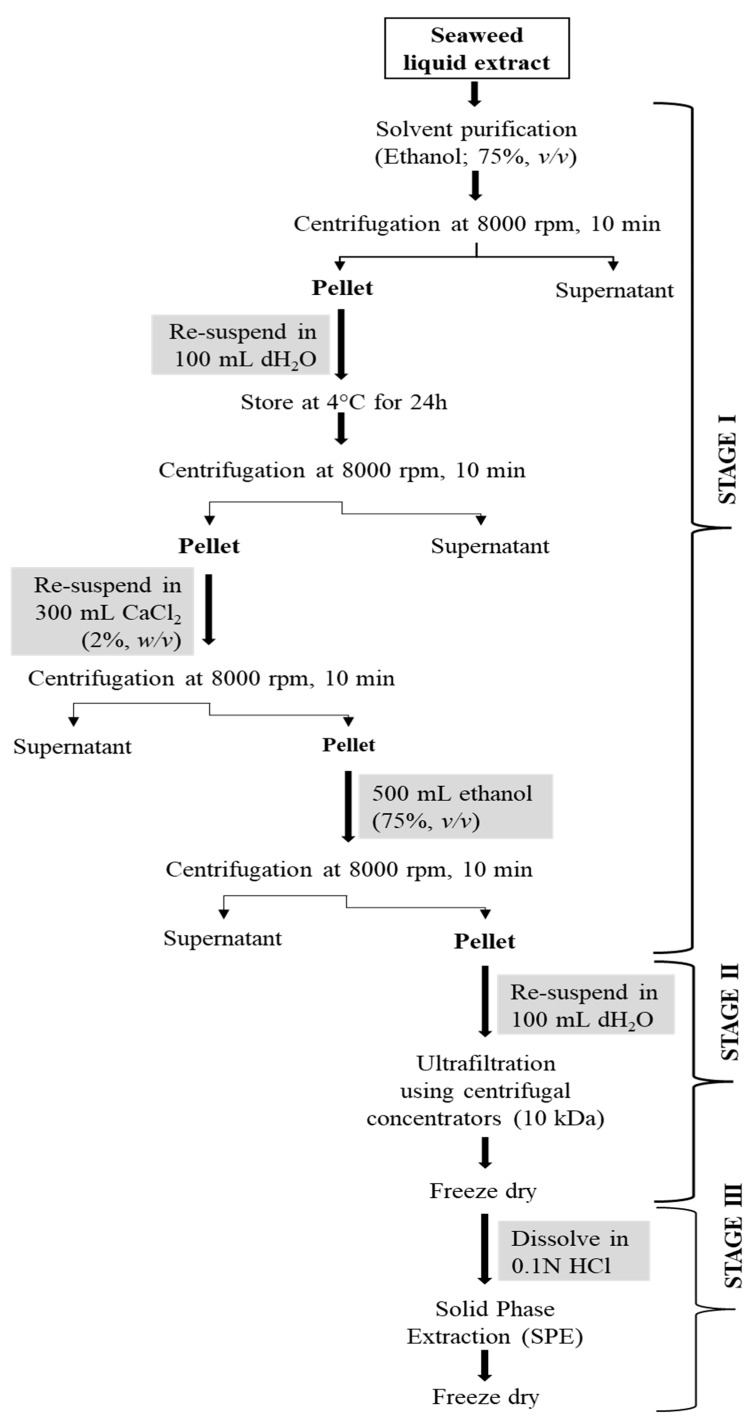
A three-step purification process for fucoidan from *A. nodosum* seaweed.

Fucoidan was purified by adopting a multi-step (three-step) protocol developed for laminarin purification earlier [23]. The steps undertaken for the purification of fucoidan from the crude extract are presented in Figure 3.

In stage 1, the *A. nodosum* liquid extract was mixed with ethanol (1:4, *v/v*) and centrifuged at 8000 rpm for 10 min following which the pellet was collected. The obtained pellet was resuspended in ultrapure water (100 mL) and stored at 4 °C for 24 h to remove protein impurities. The mixture was treated with 300 mL of CaCl_2_ (2%, *w/v*) and centrifuged at 8000 rpm for 10 min to remove alginate content. After centrifugation, the pellet was resuspended in 500 mL of ethanol and centrifuged at 8000 rpm for 10 min. The pellet was again dissolved in ultrapure water (100 mL) and submitted for stage 2 purification involving a molecular weight cut-off (MWCO) centrifugal concentrator (10 kDa cut-off; IVSS VIVASPIN, Sigma Aldrich, Ireland). The concentrated sample was freeze-dried, dissolved in 0.1M HCl, filtered using 0.45 μm syringe filters, and loaded onto a solid phase extraction (SPE) cartridge (Strata SI-1 Silica, 55 μm, 70A, 2 g/12 mL Giga Tubes, Phenomenex) for the stage 3 purification step. The collected fractions were freeze-dried and tested for polysaccharide content, structural characteristics, and in vitro antioxidant activity.

### 3.6. Quantification of Fucoidan Using HPLC

The quantification of fucoidan in purified fractions of *A. nodosum* extract was achieved on a Varian *Prostar* HPLC separation module with an integrated data system (Varian MS Workstation, version 6.9.2). The HPLC system was accompanied by an autosampler (Varian Prostar, model 450), a degasser isocratic pump, and a refractive index (RI) detector (Varian, model 350). The separation was performed at 25 °C using an ultrahydrogel™ 500 size exclusion column (7.8 × 300 mm; Waters, Herts, UK). The mobile phase consisting of ultrapure water (HPLC grade) was eluted at 0.6 mL/min in isocratic mode. The injection volume of 10 µL was kept constant for samples and standard compounds [53]. The limit of detection and the limit of quantification was found to be 0.003 mg/ml and 0.009 mg/mL, respectively. Meanwhile, the standard error for the method was 0.000167. Considering the nature of the extraction procedure (which involves 0.1N HCl solvent, 120 °C temperature for 62 min), no hydrolysis of fucoidan was performed. The concentration of fucoidan in the crude extract and purified fractions was directly determined using a calibration curve of commercial fucoidan, purchased from Sigma.

### 3.7. Characterisation of Purified Fucoidan Using Quadrupole Time-of-Flight Mass Spectrometry (Q-ToF-MS)

Structural characterisation of the purified fucoidan was conducted using Q-ToF-MS. Mass spectral data were recorded on positive ionization mode using an ESI interface on a Q-Tof Premier (Waters Corp., MA, USA) with 3.0 kV capillary voltage and 35 kV cone voltage in the mass range of *m/z* 100–5000. A purified sample dissolved in 50% acetonitrile (0.5 mg/mL) was directly infused in the ESI source at 10 µL/min. Source and desolvation temperatures were set at 150 °C and 350 °C, respectively.

### 3.8. Characterisation of Purified Fucoidan Using FTIR Spectrometry

The Fourier-transform infrared (FTIR) spectra of dried purified fucoidan fraction and fucoidan standard from *F. vesiculosus* (Sigma) were recorded to identify the possible functional groups as a variation in functional groups in the standard using a Perkin Elmer Spectrum GX FTIR (UATR) Microscope (USA). The spectra were recorded over the range 4000–400 cm^−1^ with 64 scans at a resolution of 0.3 cm^−1^ in transmission mode, and the differences were recorded.

### 3.9. Antioxidant Activity Analysis

#### 3.9.1. DPPH Radical Scavenging Activity Assay

The DPPH assay was performed according to the method standardized earlier [18]. The DPPH solution was freshly prepared for each experiment in methanol. Ascorbic acid was used as the positive control, and methanol was used as a blank. The ability to scavenge the DPPH radical was calculated using the following equation:

% DPPH inhibition = ((Abs _Blank_ ‒ Abs _Inhibitor_)/Abs _Blank_) × 100 (1)

where Abs _Blank_ is the absorbance of the DPPH solution without any test compounds and the Abs _Inhibitor_ is the absorbance of the tested samples or positive control after the reaction takes place.

#### 3.9.2. Ferric Reducing Antioxidant Power (FRAP)

Total antioxidant-reducing power of the crude extract and purified fractions was measured using FRAP assay according to the method reported by Benzie and Strain [54]. The sample was diluted to 1 mg/mL and assayed against trolox standards using a preheated FRAP reagent at 37 °C (300 mM acetate buffer, pH 3.6; 10 mM TPTZ in 40 mM HCl and 20 mM FeCl_3_·6H_2_O in the ratio of 10:1:1, *v/v/v*). The absorbance of the reaction mixture was measured at 593 nm following incubation at 37 °C for 30 min. The FRAP values were expressed as mg trolox equivalents per g (mg TE/g) of extract.

### 3.10. Statistical Analysis

All experimental trials were conducted in duplicate. Results are expressed as mean ± standard deviation. Statistical analyses were carried out using STATGRAPHICS Centurion XV software version 15.1.02 (StatPoint Technologies Inc. Warrenton, VA, USA). The significant differences between the antioxidant capacities of the crude extract and purified fucoidan fractions were determined using analysis of variance (ANOVA) followed by least significant difference (LSD) testing. Values of *p* < 0.05 were considered significant.

## 4. Conclusions

In this study, fucoidan was extracted from *A. nodosum* by employing a hydrothermal-assisted extraction procedure (120 °C, 62 min). Composition analysis of the crude extract revealed that HAE-derived crude extract contained a high amount of fucoidan (54.4%) along with laminarin and mannitol. A multi-step (three-step) purification protocol involving solvents, MWCO filter, and SPE cartridge resulted in a high recovery of fucoidan with 63.3% purity. The efficacy of the extraction and purification protocol was determined by performing an HPLC analysis of the fractions obtained from three-steps purification. Among the purified fractions, though, the SPE fraction (step 3) contained a higher amount of fucoidan, but a similar antioxidant activity was recorded in the MWCO fraction (step 2); therefore, the MWCO fraction was selected for molecular characterisation. A Q-ToF-MS analysis of the MWCO fraction confirmed ~5.4 kDa molecular weight of purified fucoidan. Purified fractions of fucoidan exhibited either higher or similar antioxidant activity than the commercial fucoidan standard but demonstrated lower antioxidant activities in comparison with the crude extract. Both MWCO and SPE proved to be efficient techniques for the purification of a polar compound such as fucoidan, but in this study, purification of fucoidan-rich crude extract with solvents followed by MWCO filter (10 kDa) was found to be more suitable. In a multi-step purification strategy, the selection of two-step purification (involving solvents followed by MWCO filter) for recovering bioactive fucoidan not only reduced the cost (by avoiding SPE cartridge and associated solvents) of operation but also the time required to perform a three-step purification. The potential of *A. nodosum* as a sustainable seaweed biomass to produce fucoidan using an optimised purification strategy for commercial production requires further investigation, considering the importance of this sulphated polysaccharide in food and pharmaceutical applications.

## Figures and Tables

**Table 1:** Biochemical composition of dried *A. nodosum* seaweed biomass [11].

Component	Composition (mg/g Dry Weight (dw) Basis)
Dry matter *	903.8 ± 0.2
Ash	233.1 ± 3.3
Protein	61.4 ± 0.1
Ether extract	33.3 ± 0.0
Total soluble sugars	136.6 ± 0.8
Total glucan	27.0 ± 0.8
Fucoidan	100.9 ± 0.9
Total polyphenols	6.7 ± 0.1

The results are expressed as mean ± standard deviation of the mean (*n* = 3). * Dry matter and moisture content were 244.3 mg/g and 755.7 mg/g (on a fresh weight basis) respectively.

**Table 2:** Biochemical composition of the crude extract obtained from *A. nodosum* seaweed using hydrothermal-assisted extraction (HAE).

Component	Composition (mg/g Dry Weight Extract)
Dry matter	22.2 ± 2.1
Fucoidan	417.6 ± 4.1
Laminarin	165.8 ± 5.3
Alginate	41.4 ± 3.2
Mannitol	96.0 ± 1.4
Crude Protein	40.3 ± 1.2
Ash	6.7 ± 0.3

The results are expressed as mean ± standard deviation of the mean (*n* = 2).

**Table 3:** Fucoidan, laminarin, and mannitol content in different purified fractions from *A. nodosum* seaweed using high-performance liquid chromatography (HPLC).

Purified Fractions	Fucoidan	Laminarin	Mannitol
	(mg/g)	(mg/g)	(mg/g)
Solvent Purified Fraction	517.1 ± 0.02 ^a^	172.2 ± 0.01 ^a^	112.5 ± 0.02 ^a^
MWCO Fraction (10 kDa)	562.3 ± 0.08 ^b^	155.7 ± 0.09 ^b^	82.1 ± 0.03 ^b^
SPE Fraction	633.2 ± 0.03 ^c^	124.2 ± 0.02 ^c^	76.5 ± 0.05 ^c^

The results are expressed in mg/g of dry seaweed extract and mean ± standard deviation of the mean (*n* = 2). Means not sharing the same letter are significantly different (LSD) at *p* < 0.05 probability level. SPE: solid phase extraction; MWCO: molecular weight cut-off filter.

**Table 4:** Antioxidant activity of the crude extract and purified fractions of fucoidan from *A. nodosum* seaweed.

Sample	Antioxidant Activity	
	DPPH Scavenging Capacity (%)	FRAP (mg TE/g)
Crude Extract	43.1 ± 0.2 ^a^	38.6 ± 0.2 ^a^
Solvent Purified Fraction	37.8 ± 0.5 ^b^	30.8 ± 0.4 ^b^
MWCO Fraction (10 kDa)	23.1 ± 0.3 ^c^	14.8 ± 0.5 ^c^
SPE Fraction	22.6 ± 0.1 ^cd^	14.5 ± 0.3 ^cd^
Fucoidan standard (from Sigma)	21.7 ± 0.6 ^d^	13.9 ± 0.7 ^d^
Ascorbic acid	35.2 ± 0.8 ^b^	—

The results are expressed as mean ± standard deviation of the mean (*n* = 2). Means not sharing the same letter in a column are significantly different (LSD) at *p* < 0.05 probability level. FRAP: expressed as mg trolox equivalent (TE)/g of dry weight (dw) extract. SPE: solid phase extraction; MWCO: molecular weight cut-off filter.

## Data Availability

The data presented in this study are available within the article.
